# Evaluation of healing progression at surgical incision sites and the use of antiseptics for enhancing post-operative survival in subyearling Chinook salmon (*Oncorhynchus tshawytscha*)

**DOI:** 10.1371/journal.pone.0288056

**Published:** 2023-07-20

**Authors:** April S. Cameron, A. Michelle Wargo Rub, Benjamin P. Sandford

**Affiliations:** 1 Ocean Associates, Incorporated, Under Contract to the Northwest Fisheries Science Center, National Marine Fisheries Service, National Oceanic and Atmospheric Administration, Hammond, Oregon, United States of America; 2 Fish Ecology Division, Northwest Fisheries Science Center, National Marine Fisheries Service, National Oceanic and Atmospheric Administration, Hammond, Oregon, United States of America; 3 Fish Ecology Division, Northwest Fisheries Science Center, National Marine Fisheries Service, National Oceanic and Atmospheric Administration, Pasco, Washington, United States of America; Tanta University Faculty of Agriculture, EGYPT

## Abstract

In an attempt to develop more effective surgical implantation methods for fish, surgical incisions typical of those made for implanting micro-acoustic transmitters into the peritoneal cavity were evaluated on a weekly basis for healing progression using a suite of metrics. Additionally, four chemicals were evaluated at concentrations commonly used in aquaculture for their ability to prevent surgical site infection and thus to promote incision healing and survival. Chemical treatments included hydrogen peroxide (25, 50, and 100 mg l^-1^), salt (10 and 30 ppt), Argentyne (1:1, Argentyne:water), and PolyAqua (1/2 tsp 36 l^-1^). For all study fish, the presence of two intact sutures seven days post-surgery (generally associated with good suturing technique) was negatively correlated with survival. A generalized linear mixed effects model indicated that suture presence, increasing tagging temperature, and the presence of foreign material on sutures decreased survival by 0.56, 0.72 and 0.60 respectively. Conversely, evidence of suture tearing and increasing fork length at tagging increased survival by 0.24 and 0.17. The antiseptic treatments tested promoted neither faster healing of surgical incisions nor higher survival for fish held for 28 days compared to a reference group and two of the chemicals may be contraindicated for prophylactic use at published doses. These findings underscore the need for researchers to adopt a decidedly cautious approach to planning and interpretation of study outcomes that rely on telemetry tagging, carefully considering the study subjects, potential effects of the techniques used, and implications of the environmental conditions experienced.

## Introduction

Aseptic techniques are practiced in human and veterinary medicine as a routine component of any surgical procedure to prevent contamination and subsequent infection [1]. More recently, such techniques have been applied in fishery research for surgical implantation of transmitters and data loggers [2–12]. However, given the difficulty of maintaining asepsis in aquatic surgical and post-operative environments, some researchers have expressed doubt that any benefit is obtained through their use [13].

In 2007 and 2008, a large tag‑comparison study to evaluate the effects of surgically implanted acoustic transmitters on yearling and subyearling Chinook salmon (*Oncorhynchus tshawytscha*) was conducted within the Columbia River (CR), USA [14]. During said study, survival and behavior of fish implanted surgically with acoustic transmitters was compared to that of those implanted with PIT-tags using an injection method. For the field component of the study, fish were released back into the CR to resume their migration after recovering from anesthetic. For surgical implantation of acoustic transmitters, aseptic technique was practiced to the extent practically possible. For example, all surgical tools were sterilized in a steam autoclave prior to the start of each tagging day. Tools were rotated and reused between surgeries within the course of a day only after they were disinfected in 70% ethyl alcohol for a minimum of 10 min and then rinsed in distilled water. Surgical needles and sutures were similarly disinfected between surgeries. Despite these practices, during 2007, tagged subyearling Chinook salmon that had been intercepted downstream after release were observed to have considerable inflammation at incision sites and some exhibited gaping wounds in the vicinity of their incision.

Based on these observations, the field study was postponed, and additional laboratory research (described here) was conducted to determine the cause(s) of these observations and to explore more effective implantation methods. Towards this end, surgical implantation surgeries were performed on run of the river subyearling Chinook salmon captured at Lower Granite Dam during their outmigration from June 3 through July 16, 2008. These fish were then transported to the Juvenile Fish Facility at Bonneville Dam where their surgical incisions were evaluated on a weekly basis for progression of healing using a suite of metrics (Table 1). Additionally, the efficacy of several chemicals (applied topically) was tested for preventing infection and/or promoting healing of the post-operative incision sites [15–17]. The underlying hypothesis was that faster and cleaner incision healing (i.e., better tissue apposition, less inflammation, and no infection) would be correlated with higher survival.

**Table 1:** List of metrics used to evaluate incision site healing for dip study fish that survived the 24‑h post‑tagging recovery period and transport to the Bonneville fish facility.

Incision site metrics for subyearling Chinook salmon (*Oncorhynchus tshawytscha*)	
Name and description	Scale and criteria
*Sutures*	
Total number of sutures present	0, 1, 2
*Knots*	
Total number of knots present†	0, 1, 2
*Suture tearing*	
Evidence of tissue torn by suture	0 = absent, 1 = present
*Foreign material*	
Evidence of a foreign material on sutures, e.g., fungus, bacterial floc, debris, etc.	0 = absent, 1 = present
*Inflammation*	
Presence of inflammation at suture entry or exit sites or between entry and exit sites	0 = none, 1 = present at either the suture entry or exit site or in between, 2 = present at two or more suture sites including the suture entry and exit sites and/or in between
*Ulceration* ^a^	
Presence of ulceration at suture entry or exit sites or between entry and exit sites	0 = none, 1 = present at either the suture entry or exit site, 2 = present at both entry and exit site, 3 = present between entry and exit, 4 = present at entry/exit sites and in between
*Incision healing*	
Degree to which incision is healed	0 = completely healed, no scar visible, 1 = healed but scar still visible, 2 = incision not healed
*Incision apposition*	
How well the two parallel sides of the incision are approximated	0 = perfect apposition on 100% of incision length 1 = >50% of incision length perfectly apposed 2 = <50% of incision length perfectly apposed
*Incision inflammation*	
Extent of inflammation along length of the incision by percentage	0 = none, 1 = inflammation on ≤25% of incision length 2 = inflammation on 26–49% of incision length 3 = inflammation on >50% of incision length

Remaining number of "throws" is not reflected here.

^a^Does not describe degree of ulceration, but only whether underlying dermis is exposed and red.

Chemical treatments tested included hydrogen peroxide (PeroxAid; Eka Chemicals, Marietta, GA), salt (Instant Ocean; Spectrum Brands, Madison, WI), Argentyne (Argent Aquaculture, Redmond, WA), and PolyAqua (Kordon, Hayward, CA) (Table 2A and 2B). These chemicals were chosen based on their current use by the aquaculture industry as either antimicrobials/parasiticides/fungicides (hydrogen peroxide and Argentyne), an osmoregulatory enhancer (salt) or a water conditioner (PolyAqua). Apart from Argentyne, the chemicals tested have not been routinely used as antiseptics during fish implantation procedures and to the authors’ knowledge, a rigorous comparison of this nature has not been conducted.

**Table 2:** a. Antimicrobial chemicals for aquatic or fishery use by product name with active ingredients, concentration/duration of recommended application, and FDA‑approved species, life stage, and usage. b. Relevant literature on fish applications by chemical.

Chemical (Active ingredients)	*Application*	*Concentration/ duration*	*FDA‑approved species/life stage/usage*
**PeroxAid** (Hydrogen peroxide)	Immersion bath	25 mg l^-1^/30 min 50 mg l^-1^/30 min 100 mg l^-1^/30 min	All/all/fungicide, parasiticide
**Instant Ocean** (Calcium & and magnesium)	Immersion bath	10 ppt/10 min 30 ppt/30 min	Non‑specified/ non‑specified/parasiticide, osmoregulatory enhancer
**Argentyne** (Poly iodine complex/sodium bicarbonate)	Topical application to incision site	1:1/0 min	Fish/eggs/antimicrobial
**PolyAqua** (Synthetic polymer)	Immersion bath	1/2 tsp 36 l^-1^/30 min	Non‑specified/non‑specified/water conditioner

Applications in fisheries literature	
**Hydrogen Peroxide**	
Bacteria and/or fungi	Adult spring Chinook salmon [18], fall Chinook salmon eggs [19], rainbow trout eggs [20]
Bacterial Gill Disease	Hatchery reared salmonids [21], rainbow trout [22]
Columnaris	Channel catfish fingerlings and walleye [23], juvenile rainbow trout [24]
Disinfection	Rainbow trout eggs [25]
Hatching and infection	Rainbow trout eggs [26]
Parasites	Hatchery reared juvenile rainbow trout [27]
Saprolegniasis	Adult spring Chinook salmon [18], channel catfish and eggs [28,29], paddlefish, walleye and white sucker eggs [30], rainbow trout and eggs [26,31]
Sea lice	Atlantic and Chinook salmon [32], farmed Atlantic salmon [33]
**Salt**	
Bacteria or fungi (eggs)	Rainbow trout [20], salmon [34]
Brown blood disease, mucus production, osmoregulation and parasites	Freshwater fish [35,36]
Flavobacterium columnare and F. psychrophilum	In vitro [37]
Handling and transport and/or therapeutic aid for freshwater disease transmission	American shad [38], Chinook salmon smolts [39], delta smelt [40], fingerling shad [41], freshwater fish [42], juvenile coho salmon and steelhead trout [43], threadfin shad [44], yearling and subyearling Chinook salmon [45]
Hatching, infection and saprolegniasis	Rainbow trout eggs [26]
**Iodine**	
Bacteria, fungi, or viruses	Fish [46,47], rainbow trout eggs [20], salmonids [48]
Chemoprophylaxis and pathogen reduction	Fish eggs [47]
Disinfection (eggs)	Atlantic halibut [49], fish [50], grouper [51], lake sturgeon [52], trout [53], northern pike and walleye [54], rainbow trout [25,55,56], salmonids [48], trout [57]
Flavobacterium columnare and F. psychrophilum	In vitro [37]
Saprolegniasis	Rainbow trout eggs [26]
**Synthetic Polymer**	
Handling and transport	Delta smelt [40]
Health aid, bruises and lacerations	Aquarium fish [58,59]
Injection point sealant, slime coat restoration and/or tagging anesthetic solution additive	Bigmouth shiner, bluegill, brook stickleback, common carp, creek chub, fathead minnow, green sunfish, Iowa darter, johnny darter, largemouth bass, longnose dace, longnose sucker, rainbow trout, sand shiner, white sucker [60], juvenile salmonids [61], pacific herring [62]
Marking recovery tank additive	Subyearling Chinook salmon [63]
Surgical work surface coating	Juvenile pacific lamprey [64], juvenile salmonids [65]

## Materials and methods

### Ethics statement

This research was conducted following the U.S. government principles for utilization and care of vertebrate animals used in testing, research, and training as described by the National Institutes of Health, Office of Laboratory Animal Welfare [66]. The care and use of experimental fish complied with the animal welfare laws, guidelines and policies of the United States and states of Oregon and Washington, including the Endangered Species Act of 1973, as amended, and lawful regulations issued thereunder which relate to threatened or endangered plant or animal species. The determination of take and handling for these research purposes was federally approved by National Oceanic and Atmospheric Administration, National Marine Fisheries Service, Authorization and Permits for Protected Species, under the Federal Columbia River Power System’s 2008 Biological Opinion. Similarly, the scientific collection permit for fish and marine and freshwater invertebrates was approved by Oregon Department of Fish and Wildlife, as was the scientific collection permit for state and/or federal threatened or endangered species of marine and freshwater fish, by Washington Department of Fish and Wildlife.

Useful information was sought after in terms of an antiseptic or chemical that might help surgical incision site healing and promote species conservation. No adverse effects were anticipated from applying the treatments tested to the incisions of study fish as all chemicals tested were chosen based on their use as therapeutics or water conditioners in the aquaculture industry. Fish behavior was monitored several times daily and mortalities were removed once daily. No fish were euthanized during the experiment due to the need to evaluate whether the treatments tested promoted incision healing and thus survival. Fish surviving to the end of the 28-d holding and examination period were ultimately released to the Columbia River alive to resume their migration.

### Study area

Fish collection took place at Lower Granite Dam near Pomeroy, Washington (Rkm 695; 46.6604°N, 117.4280°W). Lower Granite Dam is located 173 km above the confluence of the Snake and Columbia Rivers. It is one of several hydropower dams within the Columbia River Basin where it is possible to intercept and collect Chinook salmon juveniles from the river as they migrate seaward. Study fish were held for observation at the Bonneville Dam Juvenile Monitoring Facility (JMF) near North Bonneville, WA (Rkm 235; 45.6443°N, 121.9406°W). Bonneville Dam is the lowest hydropower dam on the Columbia River and is approximately 265 miles from Lower Granite Dam by truck. At the time of this study, the JMF hosted both a flow-through freshwater system as well as a closed, salt water system. Readers are referred to Wargo-Rub et al. 2020 and 2011 [14,45] for a map of the study area, a detailed description of river conditions during fish collection, and for details regarding fish transport.

### Fish collection and tagging

Migrating hatchery and wild subyearling Chinook salmon were diverted from the smolt collection facility at Lower Granite Dam (Rkm 173 on the Snake River in Washington State) over 10 dates between 2 June and 15 July, 2008 for this study. These fish were routed to a concrete raceway for holding and sorted within 12-18 h of collection.

Sorting was conducted under light anesthesia with clove oil first, followed by tricaine methanesulfonate (MS-222; [67,68]). Approximately 400 fish per day (3200 total) were selected for tagging. These fish measured at least 95 mm in fork length, had no visual sign of disease or injury, and had not been previously implanted with PIT-tags. After sorting, study fish were transferred to a 975‑L tank with flow-through river water, where they were held overnight to recover from anesthesia.

On the day after collection, study fish were anesthetized in a bath containing MS-222 in concentrations ranging from 50 to 80 mg l^-1^ [14,45]. After reaching stage‑IV anesthesia (loss of equilibrium; [69]), fish were removed from the anesthetic bath and transferred in water to a data station where they were weighed, measured, photographed, and assigned to chemical treatment groups and surgeons. Assignments were made following a predetermined rotation. This ensured that on a given day, there was equal contribution among the surgeons present to each treatment group. The goal was to tag 40 fish for each treatment group every day for ten replicate days, totaling 400 fish per treatment for the study overall (Table 3). Sample sizes were determined based on availability of fish from the parent study [14] and the number of fish four surgeons could perform surgery on within a day. Study fish were equally balanced between the replicate days and between treatments. These sample sizes granted approximately 80% power to detect a 10% difference between the treatments. The treatment groups tested are shown in Table 2A, and included hydrogen peroxide (25, 50, and 100 mg l^-1^) and salt (10 and 30 ppt), both following recommended concentrations [35,70]; Argentyne (1:1, Argentyne:water), following the recommended concentration [71]; and PolyAqua (1 tsp 37.8 l^-1^), following the manufacturer’s instructions for use [59]. A reference group subjected only to a river water bath after tagging was also included. The reference group was considered to be another treatment group–i.e., not a “control” group–and analyses herein are designed to compare eight treatments.

**Table 3:** Number of fish tagged by treatment and date.

			Peroxide (mg l^-1^)			Salt (ppt)		
Date	Reference	Argent	25	50	100	10	30	PolyAqua
**3-Jun**	40	40	38	39	41	40	40	41
**5-Jun**	40	40	39	40	40	41	40	40
**10-Jun**	40	40	41	40	40	40	40	40
**12-Jun**	40	41	40	40	40	39	40	40
**18-Jun**	40	40	40	40	40	40	40	39
**20-Jun**	40	40	40	40	40	40	40	40
**27-Jun**	40	39	40	40	40	41	40	40
**1-Jul**	40	40	40	40	40	40	40	40
**10-Jul**	40	39	40	40	40	40	40	40
**16-Jul**	41	40	40	40	40	41	39	39
**Total**	401	399	398	399	401	402	399	399

From data stations, treatment fish were transported to the assigned surgeon, transferred from their container to a surgical platform made of closed cell neoprene and maintained on an anesthetic drip over their gills of MS-222 (50 mg l^-1^) for the duration of the procedure. A 2–3 mm skin incision was made on the ventral midline of each fish just anterior to the pelvic girdle with either a 3.0-mm Micro-Unitome blade (BD Medical Supplies: Franklin Lakes, NJ), a number 10 scalpel blade, or a combination of both. The choice of blade used or combination thereof was based on surgeon preference. This incision procedure and location was typical of the procedure used to surgically implant micro-acoustic tags in juvenile salmon. However, instead of an acoustic transmitter, a PIT tag (Biomark; Boise, ID, Tx-1411SST) was inserted into the peritoneal cavity to monitor incision healing of individuals throughout the study. On average, PIT tags measured 12.48 mm in length and 2.07 mm in diameter and weighed 0.1 g in air. Each PIT tag is characterized by a unique hexadecimal code that can be scanned and read through the body wall of a tagged fish.

Incisions were closed with 2, 5-0 absorbable monofilament sutures in a simple interrupted pattern. Surgical tagging occurred simultaneously at four stations, with approximately 75-100 PIT tags implanted per hour for a total of 320 fish tagged daily. All surgical tools were sterilized in a steam autoclave prior to the start of each tagging day. All PIT tags were disinfected in 70% ethyl alcohol for a minimum of 10 min and then rinsed in distilled water prior to use. Suture material and surgical tools were disinfected and rinsed in the same manner between consecutive surgeries.

In total, 3198 subyearling Chinook salmon were tagged for this this study and subjected to one of eight treatments (including a reference group; Table 3). For the first eight replicates evaluated, eight primary surgeons implanted PIT tags in a total of 2,297 bath treatment and reference fish (range 127–451 each). These eight primary surgeons attended from two to seven replicate days, when five of the eight generally tagged ~64 fish each. Four other surgeons tagged a total of 262 fish (range 34–99 each) over the 8 replicates. Each surgeon attended 1–2 replicate tagging sessions and marked 4–66 fish.

### Treatments

Immediately after surgical tagging, fish were transferred in water to a second data station, and their incision was photographed. At this stage, all study fish except the Argentyne treatment group were placed by treatment cohort (N = 40 fish) into 5-gal buckets supplied with flow-through river water to recover from the anesthetic.

For the Argentyne treatments, a disinfectant solution of 1:1, Argentyne:water, was applied to incisions prior to placement of fish in recovery buckets. Argentyne is composed of polyvinylpyrrolidone iodine, and was applied topically, as opposed to the other treatments, which were administered via immersion bath. For bath treatments, the last fish implanted with a PIT tag was allowed to recover from anesthesia for a minimum of 10 min before netting them from recovery buckets with soft fine mesh dip nets and distributing them to their respective treatment bath. Chemical treatments were prepared by adding premeasured amounts of each chemical and 36 L of fresh river water to pans of dark blue, nonporous polyethylene measuring 66.0 × 45.7 × 25.4 cm (Polylewton; U.S. Plastic, Lima, OH). Reference and Argentyne treatment groups were subjected to baths filled with fresh river water only. There was one bath prepared for each treatment and all were aerated throughout the treatment.

Peroxide bath solutions of 25, 50, and 100 mg l^-1^ were prepared by adding 2.6, 5.1, and 10.3 mL, respectively, of 35% hydrogen peroxide to 36 L of river water. Each bath concentration was verified using semi-quantitative test strips (Quantofix Peroxide 100; Macherey-Nagel, Dueren, Germany). Strip color codes indicated concentrations of 1, 3, 10, 30, and 100 mg of peroxide l^-1^ of river water, ensuring that each bath concentration fell within the appropriate ranges of 10-30, 30-100, and 100 mg peroxide l^-1^ river water.

Salt baths were made using Instant Ocean to prepare solutions of 360 g and 1,080 g salt 10 l^-1^ river water for the respective 10 and 30 ppt treatments. Salt slurries were mixed at least 4 hours prior to use to allow for maximum dissolution. Just prior to treatment, slurries were transferred to treatment pans, and fresh river water was added to bring the entire volume up to 36 L. Salinities were then measured using a handheld refractometer, and additional solute was added in 100‑g increments as needed to bring them up to 10 and 30 ppt. Salinity levels were also verified using a multi-parameter water quality meter (h19828; Hanna Instruments, Woonsocket, RI).

Water conditioner treatment baths were prepared by adding 1/2 tsp of PolyAqua to 36 L of river water. This was lower than the recommended maximum label dose of 1 tsp PolyAqua 37.8 l^-1^ water for ease and consistency of measurement. Reference fish were exposed only to a river water bath.

For each group, treatment time started once the last fish of the group had been transferred to the bath. Treatment duration for all but the 30 salt ppt group was 30 min. The duration of the 30 salt ppt treatment was 10 min [35]. The time of entry between the first and last fish transferred for each group was less than one minute.

Temperature, pH, and dissolved oxygen (DO) were measured in each treatment bath at 0, 15, and 30 min (30 ppt salt bath, 0 and 10 min) using the Hanna meter. Total dissolved solids were also measured in the reference bath at time zero using the Hanna meter, as was total water hardness, which was measured using water quality test strips (SofChek; Hach Company, Loveland, CO). The Hanna instrument was recalibrated weekly for conductivity (80,000 μS cm^-1^), pH (3 point; 4.01, 7.01, and 10.1), and DO (% saturation), according to manufacturer’s recommendations.

Fish behavior was monitored every 5 min during treatment, with observers noting any deviations from normal swimming behavior and spatial distribution. Normal swimming behavior was indicated by calm but continuous swimming, as opposed to resting on the bottom, jumping, or gulping air at the surface. Normal spatial distribution was indicated by even distribution throughout the water column as opposed to aggregation at or near the bottom or at the surface.

Upon removal from treatment baths, fish were divided equally between one of two 75-L (19.8 gal) stainless steel holding tanks supplied with flow‑through river water. Fish remained in holding tanks before being transported by truck 12-24 h later, depending on when the truck was loaded, to the Bonneville Dam juvenile fish monitoring facility in North Bonneville, WA (Rkm 235 on the Columbia River). Mean transport time was 6 h 14 min. Jugs containing frozen river water were added to the transport tank during transports to keep water temperatures within 1°C of the temperature at departure. This method proved successful for the first four tagging dates as well as for the sixth, ninth, and tenth date. However, for the fifth, seventh, and eighth dates, temperature during transport varied from 3–5°C above that at departure from Lower Granite Dam [14]. Fish that died within 24 h of tagging as well as those that did not survive transport, were excluded from the study.

### Laboratory holding, observation, and incision evaluation

Upon arrival at the monitoring facility, fish were water-to-water transferred to 1,893‑L (500‑gal) circular tanks and held by date tagged (or replicate group) for the remainder of the study. Initial stocking density was less than 2 kg m^-3^ and remained low throughout the 28-d holding period. Study tanks were supplied with flow‑through river water from the CR, and fish were fed ad libitum, a diet consisting of a mixture of appropriately sized semi-moist pellets (BioDiet Grower; BioOregon, Hammond, OR). Holding tanks were checked daily for mortalities which were removed and recorded. All surviving fish were anesthetized, examined, and evaluated for progression of incision healing on days 7, 14, 21, and 28 post-tagging based on a suite of metrics (Table 1). During this process, right‑ and left‑lateral, full‑body photographs were taken of each fish, as well as a close‑up photograph of each incision (S1 Fig shows examples of metrics used to evaluate healing progress). After being held for 28 d, survivors were released to the river to resume migration.

### Statistical evaluations

Sample size and means for mortality by treatment at 28 d were compared among surgeons, as well as among replicates. Mortality rates were not compared to draw definitive conclusions about individual surgeons because they were not consistently present throughout the study period. Rather, attempts were made to verify that treatment comparisons were not biased by differences in survival among surgeons.

The relative effects of bath treatments were evaluated four ways. First, to assess overall survival effects, cumulative survival was compared by treatment group at day 7, 14, 21, and 28. This comparison used two-factor analysis of variance (ANOVA), where a replicate “time block” was considered as a random factor and bath treatment as a fixed factor. Although the error structure of survival data was presumed to be binomial, it was also presumed that with the samples’ sizes (n > 30 for each treatment/replicate and 10 replicates) and the bulk of the values in the range > 0.40, that the data was approximately normally-distributed. Following a significant ANOVA test, Tukey’s Honestly Significant Difference (HSD) comparison test was used, which maintained the desired significance level as there were eight treatment means [72]. For this, and subsequent analyses, a significance level of α = 0.05 was established.

Second, nonparametric Kaplan-Meier “time-to-event” curves were plotted to informally examine survival patterns over time [73,74]. Because fish were released after 28 d, data from greater than 28 d were right‑censored. Kaplan-Meier curves were plotted for replicates pooled across treatments to assess seasonal survival patterns, and for treatments pooled across replicates to assess within-study 28-d survival patterns.

Third, survival to 28 days was compared between incision metric scores measured at day 7 in a three-factor ANOVA (see first analysis above). Once again replicate “time block” was considered as a random factor, and bath treatments (eight) and incision metric score (2–3 levels) as fixed factors. As above, although the error structure of survival data was presumed to be binomial, it was also presumed that there was sufficient replication to treat the data as approximately normally-distributed. Some incision metric scores were combined to avoid small sample problems when relatively few fish were scored at those levels. Following ANOVAs indicating significant differences for fixed factors, multiple comparisons using Tukey’s HSD were conducted.

Finally, for fish that were alive at 7 d, a logistic regression mixed‑effects model (GLMM) using a logit link function for analysis of survival to 28 d was run [75]. For this model, both the tagging replicate and the surgeon who conducted the PIT tag implantation surgery were included as random effects (i.e., the model intercept was different for each replicate and surgeon), and the fixed effects included treatment, incision metrics measured at 7 d, tagging temperature, fork length, and weight.

The best model was determined using Akaike’s Information Criterion (AIC) rankings [76] in three stages. First, the best model using the “measured-at-tagging” variables (see above) was identified. Next, the 7‑d incision metrics (i.e., fungus present, total sutures present, sutures tearing, suture site inflammation, suture site ulceration, and incision healing) were modeled to see if the initial best model could be refined further. For this stage, possible variables from the nine shown in Table 1 were reduced to six. Total knots present was removed owing to its high correlation with total sutures present, and incision apposition and incision inflammation were removed because each had large numbers of missing values. Finally, interactions between included variables were modeled to check for further improvement. The R program was used for all analyses [77], and specifically the package MuMIn, R package version 1.43.15 for mixed‑effects modeling.

## Results

### Surgeon effect and environmental conditions

The number of fish tagged by each surgeon in each replicate was spread equally across treatments, so differences among surgeons did not bias comparisons of mean survival (S1 Table). However, examination of the data revealed trends in survival related to tagging personnel among both primary surgeons and those who tagged periodically throughout the study period). Marginal means for mortality ranged 28.2-54.8% among surgeons and 21.0-66.8% among replicates. Individual surgeons were not compared definitively, as they were not present consistently throughout the study. However, survival to 28 d ranked consistently among surgeons within individual replicates, thus revealing that some surgeons had higher survival than others (S2 Table).

The mean number of sutures remaining at 7 d by surgeon was also examined. This examination showed that surgeons with high suture retention (1.9-2.0 sutures), associated with more concise, secure knots/ligatures, also had lower survival at 28 d than their cohorts with lower mean suture retention (1.1-1.3 sutures; S2A and S2B Fig).

With respect to environmental conditions experienced by study fish during bath treatments, for all replicates and treatments, the DO generally increased from the beginning (mean = 9.8 mg dl^-1^; range = 7 to 19 mg dl^-1^) to the end (mean = 12.8 mg dl^-1^; range = 7.44–23.0 mg dl^-1^). For all but a few replicates and treatments, the pH dropped only slightly from the beginning (mean 7.9) to the end of treatment (mean 7.3). The pH dropped below 7.0 during replicates 6 (peroxide, salt at 10, and PolyAqua), 7 (reference, peroxide, Argentyne, and PolyAqua) and 9 (reference, Argentyne, and PolyAqua). Tagging temperature did not vary by more than 0.5°C throughout a given day. However, temperature increased from 11 to 18°C, from early to late replicates.

Isolated jumping, and some flashing, nosing, and gulping were observed in all treatment groups at some point during the study. However, there was no evidence of group behavior having been influenced by a specific treatment or environmental parameter such as acidic pH levels.

### Fish removed from the survival analyses

As previously mentioned, fish that died prior to ponding at Bonneville Dam (i.e., within the first 24 h post-tagging) were excluded from survival comparisons. This early mortality was low overall and was not equal among treatment groups (S3 Table).

Twenty-four-hour post-treatment mortality was highest for the three peroxide treatment groups, ranging 2.8‑8.2% over all replicates combined, and was less than or equal to 1.5% for all other treatments. Overall, mortality prior to ponding was highest for replicates 7 (10%) and 10 (6.6%) tagged on 27 June and 16 July, respectively, and was below 3% for all other replicates (Table 4). Notably, more than 80% of all treatment fish from replicates 9 and 10 died very soon after transfer to the monitoring facility. Therefore, fish tagged on these dates were also omitted from the survival comparisons by treatment. For the modeling analyses, only 24‑h mortalities and fish tagged for replicate 10 were omitted.

**Table 4:** Number of subyearling Chinook salmon (*Oncorhynchus tshawytscha*) surgically PIT-tagged by replicate and date along with average fork length and temperature at tagging. Table also shows the number of fish that survived transport to the Bonneville Juvenile Fish Facility (Ponded at Bonneville) and the temperature on holding day 28 corresponding to each replicate group.

Replicate	Date	Tagged (N)	Ponded at Bonneville (N)	Fork Length mm (SD)	Water Temperature (°C)	
					At Tagging	At d 28 Holding
1	3 Jun	319	319	110.3 (5.1)	11.2	17.4
2	5 Jun	320	318	105.7 (4.7)	10.2	17.6
3	10 Jun	321	320	107.3 (6.4)	11.2	18.3
4	12 Jun	320	314	115.1 (4.8)	11.1	18.7
5	18 Jun	319	318	121.1 (5.6)	13.5	19.7
6	20 Jun	320	320	109.6 (6.5)	13.1	19.4
7	27 Jun	320	289	110.7 (7.5)	14.1	19.4
8	1 Jul	320	313	105.5 (6.8)	15.7	20.0
9	10 Jul	319	310	109.7 (7.7)	17.3	20.6
10	16 Jul	320	299	108.7 (4.8)	17.8	20.7

### Survival

Percent survival by treatment and replicate at 7, 14, 21, and 28 d is detailed in Table 5. Overall, there were significant differences in survival identified at 7 d (*F* = 2.61, *P* = 0.023). However, the Tukey comparison tests showed that there were differences between replicate days but no significant differences between the bath treatments. By 14, 21, and 28 d there were no significant differences in survival identified between replicate days or between treatments (*F* = 1.74, 1.81, and 1.36; *P* = 0.122, 0.107, and 0.244). Diagnostics showed a good model fit for ANOVA, with high *R*^2^ values (range .83-.92 for the four comparisons).

**Table 5:** Average survival for subyearling Chinook salmon (*Oncorhynchus tshawytscha*) held at Bonneville Dam by treatment (all replicates combined) at 7, 14, 21, and 28 d.

	Days post-tagging			
	7	14	21	28
Treatment	Mean (SE)	Mean (SE)	Mean (SE)	Mean (SE)
**Argentyne**	0.959 (0.011)	0.804 (0.024)	0.719 (0.023)	0.619 (0.022)
**Reference**	0.952 (0.010)	0.830 (0.022)	0.737 (0.023)	0.658 (0.023)
**Peroxide 25 mg l** ^**-1**^	0.932 (0.013)	0.784 (0.024)	0.686 (0.023)	0.591 (0.023)
**Peroxide 50 mg l** ^**-1**^	0.913 (0.022)	0.784 (0.029)	0.683 (0.026)	0.598 (0.023)
**Peroxide 100 mg l** ^**-1**^	0.912 (0.013)	0.777 (0.021)	0.676 (0.022)	0.598 (0.023)
**PolyAqua**	0.918 (0.015)	0.767 (0.026)	0.700 (0.026)	0.621 (0.025)
**Salt 10 ppt**	0.950 (0.010)	0.822 (0.022)	0.756 (0.024)	0.673 (0.022)
**Salt 30 ppt**	0.971 (0.008)	0.835 (0.021)	0.741 (0.022)	0.672 (0.019)

**Fig 1:**
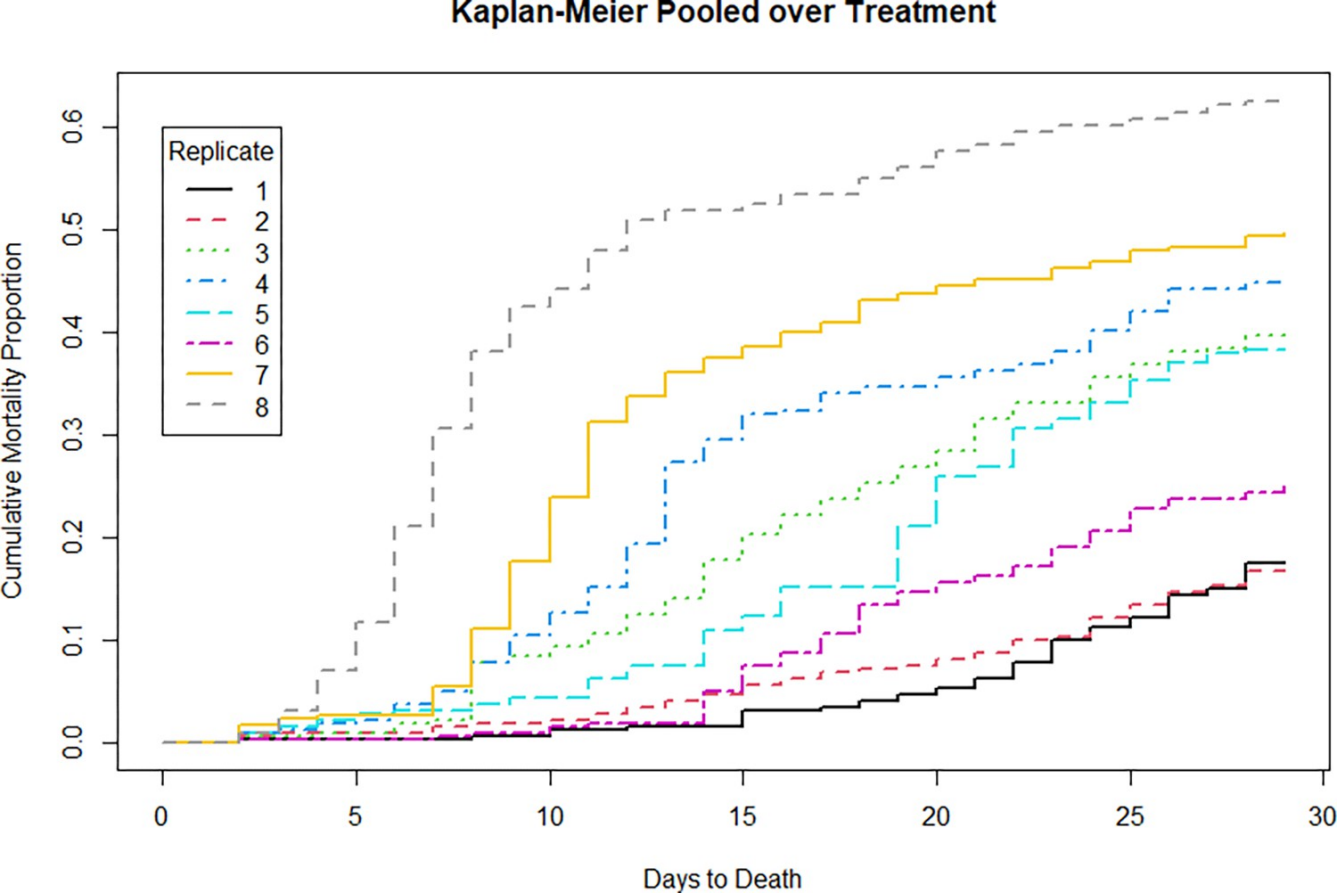
Nonparametric Kaplan‑Meier estimated mortality for replicates 1–8 pooled across treatments.

**Fig 2:**
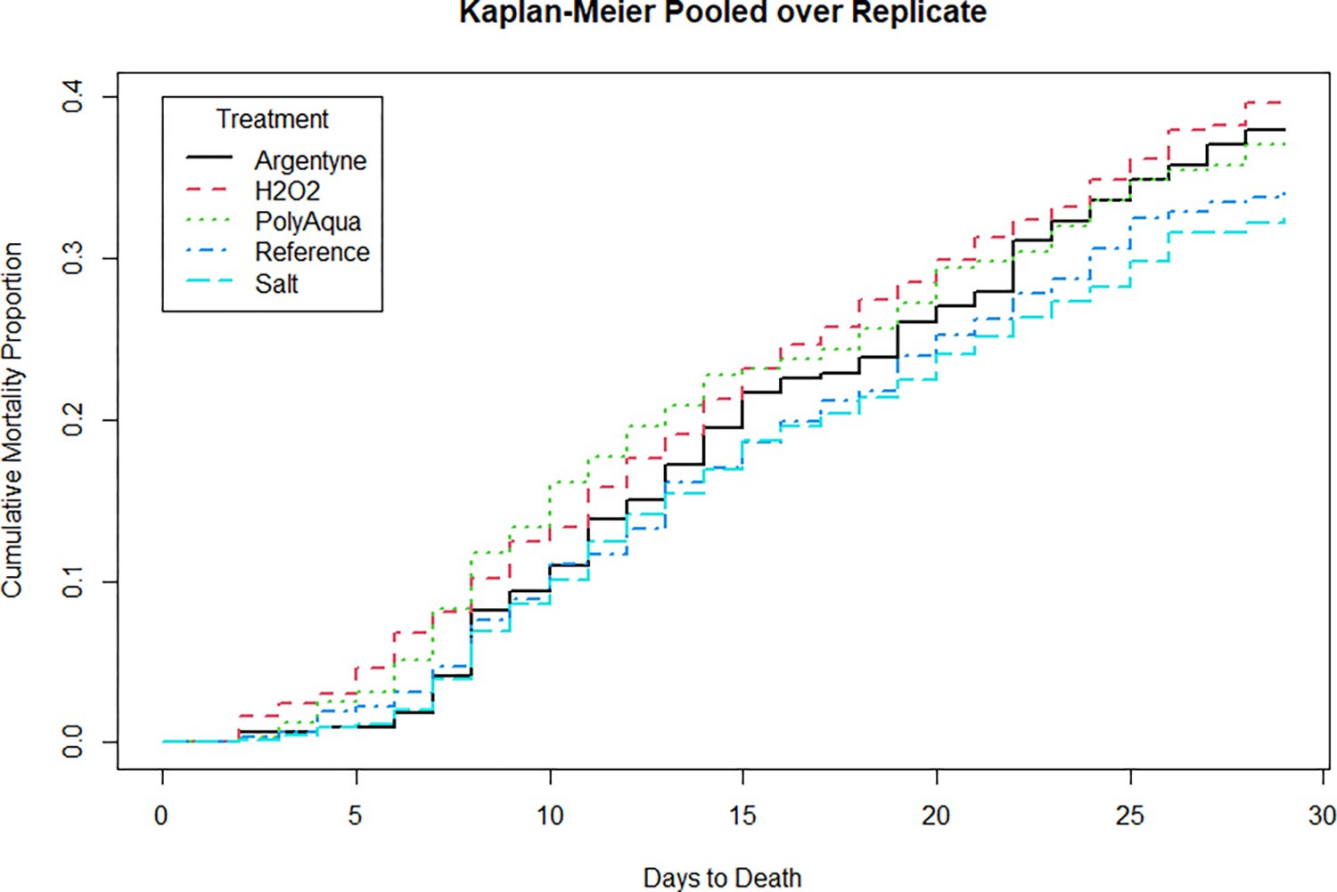
Nonparametric Kaplan-Meier estimated mortality for treatments pooled across replicates 1–8. The mortality curves for the three peroxide treatments were very similar, therefore they were pooled for this analysis. The two salt treatments were also pooled due to having very similar mortality curves.

For individual days-replicates, non-parametric Kaplan-Meier curves of mortality showed increasing patterns through time (Fig 1). This pattern was also like that observed in a long-term holding study (that was run in tandem) wherein mortality was lowest in the first two replicates and highest in the last two [14,45]. Kaplan-Meier curves describing mortality by treatment showed similar temporal patterns of somewhat constant mortality (Fig 2).

### Incision evaluations

Analysis of Variance testing indicated that there were no significant differences in survival to Day 28 among bath treatments for fish alive and scored for incision metrics on Day 7 (Table 5). As such, all results and comparisons below between levels of incision metric are averaged across treatment groups, effectively increasing replication to 8 replicates x 8 treatments (i.e., n = 64). For fish observed at 7 d with sutures intact, survival was significantly lower at 28 d (by 0.12) compared to those with only one or zero sutures intact (Tukey HSD adjusted P = 0.002 or P = 0.004, respectively). Survival for fish with one or zero sutures intact was not different (Tukey HSD adjusted P = 0.998). Evidence of suture tearing at 7 d was positively correlated with 28 d survival (P = 0.002). Fish with torn sutures had 0.08 higher survival than those without evidence of tearing (Table 5). There was no significant relationship between 28 d survival and the presence of fungus on sutures, inflammation, ulceration, or incision healing metrics as measured at 7 d (Table 6).

**Table 6:** Mean survival proportions (to 28 d) for each incision metric score as rated by examiners at 7 d for all treatments combined. Mean survival estimates are not included for bath treatments as that factor was not significant for all incision metric ANOVA tests. Incision metric score means had a maximum denominator, n = 64, (8 treatments x 8 replicate tagging days) with all incision metric levels present.

Incision Metric	ANOVA						Metric	Fish	Estimated Survival			Tukey HSD	
							Level or Score	Number	n	Mean	SE	Pair	adj *P*
Sutures Present	Factor	*Df*	*Sum Sq*	*Mean Sq*	*F*	*P*							
	Replicates	7	2.116	0.3023	6.33		0	264	58	0.758	0.038	0 vs 2	0.004
	Treatments	7	0.318	0.0454	0.95	0.469	1	316	63	0.767	0.031	1 vs 2	0.002
	Sutures Present	2	0.715	0.3574	7.48	0.001	2	1678	64	0.636	0.022	1 vs 0	0.998
	Residuals	168	8.023	0.0478									
Fungus Present	Factor	*Df*	*Sum Sq*	*Mean Sq*	*F*	*P*							
	Replicates	7	1.768	0.2526	5.09		0	2050	64	0.694	0.016		
	Treatments	7	0.207	0.0295	0.60	0.759	1	208	50	0.611	0.048		
	Fungus Present	1	0.108	0.1081	2.18	0.143							
	Residuals	98	4.868	0.0497									
Suture Tearing	Factor	*Df*	*Sum Sq*	*Mean Sq*	*F*	*P*							
	Replicates	7	2.574	0.3678	15.14		0	981	64	0.612	0.031		
	Treatments	7	0.146	0.0208	0.86	0.544	1	1277	64	0.699	0.020		
	Sutures Tearing	1	0.242	0.2424	9.98	0.002							
	Residuals	112	2.720	0.0243									
Inflammation	Factor	*Df*	*Sum Sq*	*Mean Sq*	*F*	*P*							
(suture site)	Replicates	7	2.004	0.2863	6.54		0	1152	59	0.703	0.022		
	Treatments	7	0.097	0.0138	0.32	0.946	1	451	64	0.673	0.031		
	Inflammation	2	0.019	0.0096	0.22	0.804	2 or 3	601+54	62	0.666	0.031		
	Residuals	168	7.359	0.0438									
Ulceration	Factor	*Df*	*Sum Sq*	*Mean Sq*	*F*	*P*							
(suture site)	Replicates	7	2.812	0.4017	6.68		0	1644	64	0.675	0.020		
	Treatments	7	0.247	0.0352	0.59	0.767	1	185	57	0.690	0.042		
	Ulceration	2	0.020	0.0102	0.17	0.844	2 or 3	328+101	60	0.663	0.040		
	Residuals	164	9.862	0.0601									
Incision Healing	Factor	*Df*	*Sum Sq*	*Mean Sq*	*F*	*P*							
	Replicates	7	2.293	0.3276	7.95		0 or 1	376+22	59	0.647	0.040		
	Treatments	7	0.144	0.0205	0.50	0.834	2	1860	64	0.676	0.018		
	Incision Healing	1	0.053	0.0527	1.28	0.261							
	Residuals	107	4.412	0.0412									

### Survival modeling

For fish alive at 7 d, the first stage in modeling 28‑d survival showed that the best model included only tagging temperature and fork length at tagging (Table 7A and 7B). The next three best models were between 1 and 2 AIC units larger, and all included fork length. One was only fork length and the other two included weight, which was quite correlated with fork length (*r* = 0.76) and thus adding minimal information. Therefore, the model that included only temperature and fork length was used in further modeling.

**Table 7:** a. Mixed Linear Effects modeling results (GLMM, using a logit link function) for 28 d survival. Random-effects covariates included in all models were replicate tagging days and surgeons. Fixed-effect covariates tested against survival were: Treatment, fork length, weight, water temperature at tagging, presence of foreign material (0,1), sutures (0,1,2), suture tearing (0,1), inflammation or ulceration at suture site (0,1,2), and healing at suture site (1,2). See Table 1 for the full list of post-incision metrics. For 28-d survival we evaluated models at three stages: Variable at time of tagging, incision metrics after 7d for survivors, and 2 –way interactions among covariates. Only models with delta AIC values within approximately 2 units from the best model were included in the table, although the process included all subsets as possible models. b. Final GLMM linear mixed model.

Stage and Terms		Df	AIC	Delta AIC	Weight
**Tagging**					
Length + temperature		5	2736.560	0.00	0.26
Length + temperature + weight		6	2737.612	1.05	0.16
Length + weight		5	2737.879	1.32	0.14
Length		4	2737.988	1.43	0.13
Temperature + weight		5	2738.718	2.16	0.09
**Incision metrics at 7 d (All models included length and temperature)**					
Foreign material + sutures + tearing		9	2711.696	0.00	0.38
Foreign material + sutures + incision healing + tearing		10	2713.649	1.95	0.14
Foreign material + inflammation + sutures		8	2713.816	2.12	0.13
**Interaction (All models included length + temperature + foreign material + sutures + tearing)**					
Length x foreign material and x tearing		11	2710.589	0.00	0.22
Length x foreign material		10	2711.517	0.93	0.14
No interaction		9	2711.696	1.11	0.13
Length x temperature		10	2711.708	1.12	0.13
Length x foreign material, and x tearing, and x temperature		12	2711.897	1.31	0.12
Length x tearing		10	2712.354	1.77	0.09
Temperature x tearing		10	2712.599	2.01	0.08
				**95% Profile**	**Odds**
**Parameter**	**Estimate**	**SE**	**Conf Int**	**Ratio**	**95% Conf Int**
Intercept	1.213	0.253	(0.711, 1.727)		
Temperature	-0.329	0.133	(-0.613, -0.039)	0.72	(0.54, 0.96)
Fork Length	0.159	0.057	(0.048, 0.271)	1.17	(1.05, 1.31)
Foreign material present	-0.515	0.165	(-0.838, -0.192)	0.60	(0.43, 0.83)
Sutures Present (1)	-0.037	0.210	(-0.449, 0.375)	0.96	(0.64, 1.46)
Sutures Present (2)	-0.585	0.200	(-0.981, -0.197)	0.56	(0.38, 0.82)
Suture Tearing	0.214	0.106	(0.007, 0.422)	1.24	(1.01, 1.52)

The second stage of modeling showed the best model included tagging temperature and fork length as well as total sutures present, foreign material present, and suture tearing (Table 7A and 7B). Although a couple of models that included ulceration or inflammation had delta AIC values below 2, they were expanded versions of the best model and did not add significant information.

From the third stage evaluation of interactions between the variables included in the stage two best model, seven combinations of interaction terms, including none, were plausible, with ΔAIC values between 1 and 2. However, none of the interaction models substantially improved the stage two best model, so following the principle of parsimony [76], the no-interaction model was chosen as the best model (Table 7A and 7B). All of the models accounted for random effects of replicate tagging days and surgeon. It is important to note that none of the best models included bath treatment. This is because it was not identified as a significant factor when tested with fork length and water temperature at tagging. As such, similar to ANOVA testing, modeling did not identify significant differences in survival related to bath treatment.

**Fig 3:**
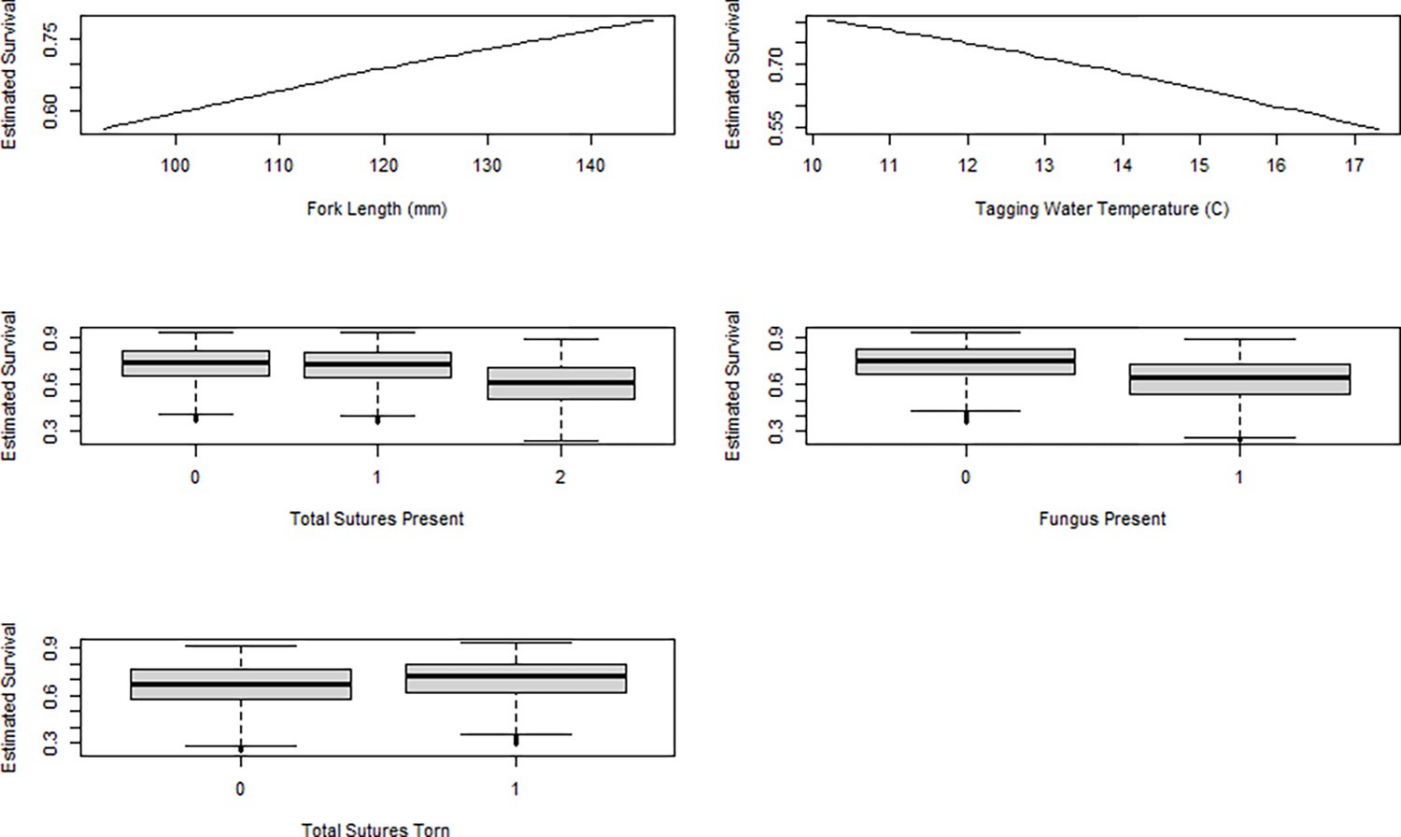
Boxplots showing levels of explanatory variables determined to significantly explain survival to 28 d for fish that survived 7 d and had recorded metrics on surgical wounds. Results are for estimated fitted values from a linear mixed effects model evaluated by AIC. Boxplots of the fitted estimated survival values show median values surrounded by a box of the 25^th^ to 75^th^ percentiles (i.e. interquartile range) with whiskers at the outlying values, which are 1.5 times the interquartile range extended from the median.

Overall estimated 28 d survival for fish that were alive and observed at 7 d increased from 0.60 to 0.75 as fork length increased from 100 to 140 mm (Fig 3). Conversely, estimated survival decreased from about 0.77 to 0.60 as water temperature at tagging increased from 11 to 16 C. For fish with one or zero sutures remaining at 7 d post-tagging, estimated 28 d survival for fish alive and observed at 7 d averaged around 0.75, but this estimate decreased to 0.60 for fish with two sutures present. When foreign material was not present at the incision site at 7 d, estimated 28 d survival averaged 0.75. In comparison, estimated average survival was only 0.65 when foreign material was present. Finally, when there was no tearing observed at the suture site on day 7, survival averaged 0.63. However, when one or two sutures were observed to be tearing through the dermis, estimated survival was higher at 0.67.

## Discussion

This study set out to evaluate the healing progression of surgical incisions in subyearling Chinook salmon. Motivated by observing incision dehiscence in migrating fish surgically tagged the previous year, the main goal was to gain insight into the etiology behind these incision failures. An additional goal was to identify a treatment that might promote healing.

This study found that mortality in some study fish was likely related to retained sutures, which had sometimes served as a nidus for fungal growth. However, despite this finding, there was no demonstrated benefit from any of the treatments tested, including those cited for their anti-fungal and anti-bacterial properties. Notably, some of the treatments tested may be contraindicated for prophylactic use at the temperatures tested.

Although there were no statistical differences in survival observed among the treatment groups, fish exposed to hydrogen peroxide at all concentrations tested, consistently exhibited lower survival than the other treatments beginning at 24 h post-treatment. Toxic effects have been observed in other studies that exposed finfish to hydrogen peroxide at similar or higher concentrations for the purpose of preventing and treating fungal infection [29,31]. In these studies, as here, toxicity increased with increasing water temperature. Based on these observations, and despite its low regulatory priority by the U.S. Food and Drug Administration (FDA), extreme caution is warranted when subjecting finfish to hydrogen peroxide submersion at therapeutic levels.

Furthermore, despite the widespread use of PolyAqua, and similar products that are promoted as health aids for bruised and lacerated fish, no evidence was found that it improved survival. It should be noted that very little is actually known about the effectiveness and safety of PolyAqua and similar products [78], and that although the difference in survival was not statistically significant between reference fish and treatment fish exposed to PolyAqua, the latter had lower survival.

Salt is also considered a low regulatory priority substance by the FDA [7] and is used in aquaculture as an inexpensive, readily available therapeutic. During our study of tag‑effects on yearling and subyearling Chinook salmon [14,45], fish were transferred from freshwater to artificial seawater on holding day 15 (35 ppt; 11-13°C). After transfer, these study fish experienced a decline in their rate of post-tagging mortality, indicating that the addition of salt may have been beneficial. However, during the current study, short-term post-operative salt baths did not significantly improve survival for treatment fish compared to the reference group.

Similarly, a one-time application of Argentyne to the surgical incisions of treatment fish did not improve survival or promote healing relative to reference fish.

Failure to impart a significant effect on wound healing or subsequent fungal growth may have been due to the relatively short duration of contact with Argentyne. Application times recommended for disinfecting eggs are significantly longer, at 10-15 minutes, and this treatment is typically repeated through time [48-57]. In a human surgical application, one study determined that a minimum contact time of 3 minutes was necessary for maximum disinfection of hands prior to conducting surgical procedures [79]. However, keeping fish out of water longer to prolong application time or actually immersing fish in Argentyne were both options expected to cause more harm than good.

The presence of two secure ligatures at 7 d post-tagging was detrimental to fish survival through 28 d, based on comparisons of survival through time and on observations of the incision healing process. In some fish, the suture material also provided a nidus for fungal growth (also correlated with higher fish mortality) and ulcers were commonly observed beneath the suture material. Mechanistically, bacteria and/or fungi may have penetrated the dermal layer through these ulcers, leading to sepsis and subsequent death. No infectious agents were identified during the tag effects study [14] when inriver‑migrant fish were recaptured after surgery and evaluated for gross and histological changes. However, of the subyearling Chinook salmon surgically implanted in 2007, only nine of 100 targeted were recaptured for necropsy. This dearth of subyearling recaptures was due to high mortality overall, so presumably, fish that were recaptured were the most robust of the tagged cohort.

Although this study found suture presence at seven days post-surgery was negatively correlated with survival, it is important to note that sutures can play an integral role in the healing process of surgical wounds in that they can neutralize tension at the incision site and promote tissue apposition. Booth (1985) described the ideal suture material as easy to handle and able to "maintain adequate tensile strength until its purpose is served". He further defined the ideal suture material as "nonelectrolytic, noncapillary, nonallergenic, and noncarcinogenic" [80].

For this study, properly tied sutures were expected to remain intact for approximately 2 weeks, the length of time anticipated for surgical incisions to functionally heal. Indeed, a respective 33.0 and 20.7% of incisions were not yet healed at 7 and 14 days post-surgery. This observation supports the need for suture presence beyond 7d to ensure optimal healing. Based on these findings, mitigation of suture impacts remains an important challenge.

In some cases, one can forego the use of sutures altogether; however, significant rates of tag loss may occur in study fish with no incision closure. Depending on the length, incisions without closure might also gape, thus exposing the body cavity to environmental pathogens or simply delaying healing due to poor tissue apposition. Braided or natural suture material may be chosen for its ability to break down faster than synthetic monofilament material, but these materials can potentially "wick" water and foreign material into the muscle layer [81–87]. Incision closure with alternative materials such as surgical glue carries a different suite of risks. Such closures can result in the incision reopening prematurely, can serve as a physical barrier to healing, and can lead to the formation of granulomas or fistulae [88–91].

Another option, which at first might seem counterintuitive, would be to use non-absorbable nylon to close surgical tag incisions. Non‑absorbable nylon is characterized as having poor knot security relative to other suture material. As such, it tends to unravel and pull out. The authors have regularly observed this outcome, however, further study is needed before non‑absorbable nylon can be considered as a preferred suture material. Such studies would include testing to determine the number of throws or extra knots needed in addition to the first knot placed in order to retain the ligature for an optimal period. This period would be long enough to promote healing but not long enough to serve as a nidus for foreign material. Future testing might also explore the use of one or more granny knots, which are prone to slippage, in lieu of standard square knots to secure each ligature.

The correlation between suture presence and the presence of fungi or fish mortality is increased when water temperatures exceeded 13.5°C. Not surprisingly, overall survival and healing throughout the study was strongly and negatively correlated with increasing water temperature. For replicates 9 and 10, ambient river temperature (and thus handling and tagging temperature) exceeded 17°C, and early post-tagging mortality was so high among all treatment replicates that none of them could be used in comparisons of survival at 28 d.

Other researchers have described warmer temperatures associated with delayed healing and inflammation at the incision site for surgically tagged fish [92,93]. These observations, along with those described here, serve as a reminder that surgical tagging of temperature‑sensitive species at relatively high water temperatures may create significant and potentially lethal delayed effects. For Columbia River Chinook salmon, the reported temperature threshold for injecting PIT tags is 15°C [94,95]. The results presented here indicated that an even lower maximum temperature threshold should be observed for surgical implantation of these fish.

For this study, Lower Granite Dam was used as the surgery site because of its suitability as a collection location for river‑run fish, as well as its proximity to the Columbia River hydro-system, which was the primary study area. Unfortunately, the high temperatures at this location proved detrimental to survival during the river migration period of subyearling Chinook. To avoid seasonal temperature impacts in the future, implantation surgeries could either be conducted earlier in the year or in a laboratory or hatchery setting under more controlled environmental conditions. Surgically tagged fish could be held during most or all of the incision healing period, and their sutures could be removed prior to release. Of course, this practice could also confound the assumption that study fish were representative of their untagged cohorts.

## Conclusion

Electronic transmitters have allowed researchers to study survival and movement of a variety of fish in situ since the mid-1950s [96]. However, for the validity of information collected through these types of studies, it is necessary to know that the behavior and survival of tagged fish is similar to that of their untagged cohorts. One way to approach this is through a paired release study such as described in tag effects study [14]. Laboratory studies can provide useful information on critical factors such as rates of tag loss, as well as providing insight into the healing process. However, readers are cautioned that results from laboratory studies can significantly underestimate the effects of surgical tagging compared to what may be actually observed in the field [14].

Comprehensive studies such as those described and referenced here will not be feasible for most situations where telemetry is used. Therefore, it is recommended that researchers and managers adopt a decidedly cautious approach to planning and interpretation of study outcomes that rely on telemetry tagging. Researchers will be well served to lean on the information collected and insights gained from previous studies [11,12,97], particularly those that have evaluated similar species, life stages, and environmental conditions.

The results of the study presented here specifically call into question the validity of data derived from surgical tagging of temperate species at temperatures greater than 13°C. They also highlight the complications that can arise from the surgical process itself, such as retained sutures that may provide a nidus for fungi (or other foreign material). Unfortunately, an effective treatment that could be implemented during surgical tagging to prevent or mitigate these effects was not found. For fish migrating in cooler temperatures or reaching the ocean sooner, these surgical complications might never have occurred.

However, given similar conditions, the only feasible options that remain are tagging fish earlier in the migration season, tagging at locations where environmental conditions can be better controlled, foregoing the use of sutures altogether (i.e., opt for an injectable tag or a very small incision), or use suture material that has less knot security such as nylon.

## Supporting information

S1 Fig Examples of select metrics used to evaluate progression of healing including a.) suture tearing b.) presence of foreign material c.) inflammation associated with suture entrance/exit sites d.) ulceration at suture entrance/exit sites e.) incision apposition <50% of length, and f.) inflammation associated with >50% of incision length.
(TIF)

S2 Fig Photos from a surgeon with low survival relative to cohorts (a) and a surgeon with high survival relative to cohorts (b). Both photos were taken at the 7-d exam. Note the knots tied by the surgeon with low survival have remained intact over the first 7 d, as would be expected with proper knot tying technique. In comparison, knots tied by the surgeon with higher relative survival have loosened, with sutures pulling free from the incision. Photo b also illustrates suture tearing and the resulting wound perpendicular to the incision.
(TIF)

S1 Table Number of fish tagged by surgeon and replicate. The percentage of fish that died before 28 d is in parentheses.
(DOCX)

S2 Table Rank among surgeons based on percentage mortality for study replicates 1–8. Mean and overall rank scores are also listed; a lower ranking corresponds with higher mortality.
(DOCX)

S3 Table Number of fish that died prior to being ponded at the Bonneville Dam juvenile fish monitoring facility, by treatment and tagging date (%). (DOCX)

## References

[1] Mangram AJ, Horan TC, Pearson ML, Silver LC, Jarvis WR. Guideline for prevention of surgical site infection. Am J Infect Control. 1999;27(2):97–34. 10196487

[2] National Research Council. Guide for the care and use of laboratory animals. 8th ed. Washington D.C.: The National Academies Press; 1996.

[3] Chittick E. Basic fish surgery I. In: Small animal and exotics. Proceedings of the North American Veterinary Conference, Volume 19; 2005 Jan 8–12; Orlando, Florida. Gainesville: Eastern States Veterinary Association; 2005. p. 1153–5.

[4] Thorsteinsson V. Tagging methods for stock assessment and research in fisheries. Report of Concerted Action FAIR CT.96.1394 (CATAG)(IS), Marine Research Institute; 2002. Report No.: 79.

[5] Cockshutt J. Principles of surgical asepsis. In: Slatter DH, editor. Textbook of small animal surgery. 3rd ed. Philadelphia: Saunders; 2003;1. p. 149–54.

[6] Mulcahy DM. Surgical implantation of transmitters into fish. ILAR J. 2003;44(4):295–06. doi: 10.1093/ilar.44.4.295 13130160

[7] American Fisheries Society. Guidelines for the use of fishes in research. Bethesda (MD): American Fisheries Society Press; 2014.

[8] Harms CA. Surgery in fish research: common procedures and postoperative care. Lab Anim. 2005;34(1):28–34. doi: 10.1038/laban0105-28 19795589

[9] Herbst LH, Jacobson ER. Recommendations for activities involving brief captivity with non-invasive or minimally invasive procedures. Gainesville (FL): University of Florida Cooperative Marine Turtle Tagging Program; 2006 [cited 2017 April 15] Available from: https://accstr.ufl.edu/resources/tagging-program-cmttp/recommendations-for-activities-involving-brief-captivity-with-non-invasive-or-minimally-invasive-procedures/.

[10] Dvorak G. Disinfection 101. Ames (IA): The Center for Food Security and Public Health, Iowa State University; 2008.

[11] Liedtke TL, Wargo-Rub AM. Techniques for telemetry transmitter attachment and evaluation of transmitter effects on fish performance. In: Adams NS, Beeman JW, Eiler JH, editors. Telemetry techniques: a user guide for fisheries research. Bethesda (MD): American Fisheries Society; 2012. p. 45–87.

[12] Wargo-Rub AM, Jepsen N, Liedtke TL, Moser ML, Scott Weber III EP. Surgical insertion of transmitters and telemetry methods in fisheries research. Am J of Vet Res. 2014;75(4):402–16. 24669927 10.2460/ajvr.75.4.402

[13] Jepsen N, Boutrup TS, Midwood JD, Koed A. Does the level of asepsis impact the success of surgically implanting tags in Atlantic salmon? Fish Res. 2013;147:344–8.

[14] Wargo Rub AM, Sandford BP, Butzerin JM, Cameron AS. Pushing the envelope: micro-transmitter effects on small juvenile Chinook salmon (*Oncorhynchus tshawytscha*). PLOS ONE. 2020;15(3):e0230100. Available from: 10.1371/journal.pone.0230100. 32210429 PMC7094837

[15] Willoughby LG. Fungi and fish diseases. Stirling: Pisces Press; 1994.

[16] Lilley JH, Inglis V. Comparative effects of various antibiotics, fungicides and disinfectants on Aphanomyces invaderis and other saprolegniaceous fungi. Aquac Res. 1997;28(6):461–9.

[17] Noga E. Fish disease: diagnosis and treatment. Ames: Iowa State University Press; 2000.

[18] Fitzpatrick MS, Schreck CB, Chitwood RL. Technical notes: evaluation of three candidate fungicides for treatment of adult spring Chinook salmon. Prog Fish Cult. 1995;57(2):153–55.

[19] Barnes ME, Stephenson H, Gabel M. Use of hydrogen peroxide and formalin treatments during incubation of landlocked fall Chinook salmon eyed eggs. N Am J Aquac. 2003;65(2):151–4.

[20] Wagner EJ, Arndt RJ, Billman EJ, Forest A, Cavender W. Comparison of the efficacy of iodine, formalin, salt, and hydrogen peroxide for control of external bacteria on rainbow trout eggs. N Am J Aquac. 2008;70(2):118–27.

[21] Rach JJ, Gaikowski MP, Ramsay RT. Efficacy of hydrogen peroxide to control mortalities associated with bacterial gill disease infections on hatchery-reared salmonids. J Aquat. 2000;12(2):119–27.

[22] Lumsden JS, Ostland VE, Ferguson HW. Use of hydrogen peroxide to treat experimentally induced bacterial gill disease in rainbow trout. J Aquat. 1998;10(3):230–40.

[23] Rach JJ, Schleis SM, Gaikowski MP, Johnson A. Efficacy of hydrogen peroxide in controlling mortality associated with external columnaris on walleye and channel catfish fingerlings. N Am J Aquac. 2003;65(4):300–5.

[24] Speare DJ, Arsenault GJ. Effects of intermittent hydrogen peroxide exposure on growth and columnaris disease prevention of juvenile rainbow trout (*Oncorhynchus mykiss*). Can J Fish Aquat Sci. 1997;54(11):2653–8.

[25] Wagner EJ, Oplinger RW, Arndt RE, Forest AM, Bartley M. The safety and effectiveness of various hydrogen peroxide and iodine treatment regimens for rainbow trout egg disinfection. N Am J Aquac. 2010;72:34–42.

[26] Marking LL, Rach JJ, Schreier TM. American Fisheries Society Evaluation of antifungal agents for fish culture. Prog Fish Cult. 1994;56(4):225–31.

[27] Rach JJ, Gaikowski MP, Ramsay RT. Efficacy of hydrogen peroxide to control parasitic infestations on hatchery reared fish. J Aquat. 2000;12(4):267–73.

[28] Rach JJ, Valentine JJ, Schreier TM, Gaikowski MP, Crawford TG. Efficacy of hydrogen peroxide to control saprolegniasis on channel catfish (*Ictalurus punctatus*) eggs. Aquac. 2004;238(1-4):135–42.

[29] Howe GE, Gingerich WH, Dawson VK, Olson JJ. Efficacy of hydrogen peroxide for treating saprolegniasis in channel catfish. J Aquat. 1999;11(3):222–30.

[30] Gaikowski MP, Rach JJ, Drobish M, Hamilton J, Harder T, Lee LA et al. Efficacy of hydrogen peroxide in controlling mortality associated with saprolegniasis on walleye, white sucker and paddlefish eggs. N Am J Aquac. 2003;65(4):349–55.

[31] Rach JJ, Howe GE, Schreier TM, Redman SD. Effects of species, life stage, and water temperature on the toxicity of hydrogen peroxide to fish. Prog Fish Cult. 1997;59(1):41–6.

[32] Johnson SC, Constible JM, Richard J. Laboratory investigations on the efficacy of hydrogen peroxide against the salmon louse *Lepeophtheirus salmonis* and its toxological and histopathological effects on Atlantic salmon Salmo salar and Chinook salmon *Oncorhynchus tshawytscha*. Dis Aquat Org. 1993;17:197–04.

[33] Treasurer J, Grant A. The efficacy of hydrogen peroxide for the treatment of farmed Atlantic salmon, Salmo salar L. infested with sea lice (Copepoda: Caligidae). Aquac. 1997;148(4):265–5.

[34] vEdgell R, Lawseth D, McLean WE, Britton EW. The use of salt solutions to control fungus (*Saprolegnia*) infestations on salmon eggs. Prog Fish Cult. 1993;55:48–52.

[35] Francis-Floyd R. The use of salt in aquaculture. Gainesville (FL): University of Florida Institute of Food and Agricultural Sciences Extension Service; 1995 Fact Sheet No: VM86.

[36] Francis-Floyd R. Bath treatment for sick fish. Gainesville (FL): University of Florida Institute of Food and Agricultural Sciences Extension Service; 1996 Fact Sheet No: VM78.

[37] Mainous ME, Kuhn DD, Smith SA. Efficacy of common aquaculture compounds for disinfection of Flavobacterium columnare and F. psychrophilum. J Appl Aquac. 2012;24(3):262–70.

[38] Chittenden ME Jr. Transporting and handling young American shad. NY Fish Game J. 1971;18:123–8.

[39] Long CW, McComas JR, Monk BH. Use of salt (NaCl) water to reduce mortality of Chinook salmon smolts, *Oncorhynchus tshawytscha*, during handling and hauling. Mar Fish Rev. 1977;39(7):6–9.

[40] Swanson C, Mager RC. Use of salts, anesthetics, and polymers to minimize handling and transport mortality in delta smelt. 1996;125(2)326–9.

[41] Skyes JE. A method of transporting fingerling shad. Prog Fish Cult. 1950;12(3):153–9.

[42] Hattingh J, LeRoux Fourie F, van Vuren JHJ. The transport of freshwater fish. J Fish Biol. 1975;7(4):447–9.

[43] Wedemeyer G. Some physiological consequences of handling stress in the juvenile coho salmon (*Oncorhynchus kisutch*) and steelhead trout (*Salmo gairdneri*). J Fish Res Board Can. 1972;29:1780–3.

[44] Collins JL, Hulsey AH. Hauling mortality of threadfin shad reduced with M.S. 222 and salt. Prog Fish Cult. 1963;25(2):105–6.

[45] Wargo-Rub AM, Sandford BP, Gilbreath LG, Myers MS, Peterson ME, Charlton LL et al. Comparative performance of acoustic-tagged and passive integrated transponder-tagged juvenile Chinook salmon in the Columbia and Snake Rivers, 2008. Seattle (WA): Northwest Fisheries Science Center, Fish Ecology Division; 2011 Report. Contract No.: W66QKZ60441152. Sponsored by U.S. Army Corps of Engineers, Portland District.

[46] Nelson NC. A review of the literature on the use of Betadine in fisheries. Springfield (VA): National Technical Information Service; 1974 NTIS No.: PB-235 443/AS.

[47] Snieszko SF. Control of fish diseases. Mar Fish Rev Paper 1301. 1978;40(3):65–8.

[48] Amend DF, Pietsch JP. Virucidal activity of two iodophors to salmonid viruses. J Fish Res Board Can. 1972;29(1):61–5.

[49] Bergh O, Jelmert A. Iodophor disinfection of eggs of Atlantic halibut. J Aquat. 1996;8(2):135–45.

[50] Moretti A, Fernandez-Criado MP, Cittolen G, Guidastri R. Fish eggs weighing, disinfecting and counting operations. In: Manual on hatchery production of seabass and gilted seabream. Rome (IT): Food and Agriculture Organization (FAO) of the United Nations; 1999;1. p. 162–3.

[51] Tendencia EA. Effect of iodine disinfection on the bacterial flora and hatching rate of grouper, Epinephelus coioides, eggs at the cleavage and eyed stages. Bull Eur Assoc Fish Pathol. 2001;21(4):160–3.

[52] Bouchard HJ, Aloisi DB. Investigations in concurrent disinfection and de-adhesion of lake sturgeon eggs. N Am J Aquac. 2002;64(3):212–6.

[53] Gee LL, Sarles WB. The disinfection of trout eggs contaminated with Bacterium salmonicida. J Bacteriol. 1941;44(1):111–26. 10.1128/jb.44.1.111-126.1942 PMC373654 16560540

[54] Tuttle-Lau MT, Phillips KA, Gaikowski MP. Evaluation of the efficacy of iodophor disinfection of walleye and northern pike eggs to eliminate viral hemorrhagic septicemia virus. La Crosse (WI): Department of the Interior, U.S. Geological Survey; 2010 Fact Sheet No.: 2009-3107.

[55] Amend DF. Comparative toxicity of two iodophors to rainbow trout eggs. Trans Am Fish Soc. 1974;103(1):103–8.

[56] Piper RG, McElwain IB, Orme LE, McCraren JP, Fowler LG, Leonard JR. Fish hatchery management. Washington, D.C.: U.S. Department of the Interior, Fish and Wildlife Services; 1986.

[57] McFadden T. Effective disinfection of trout eggs to prevent egg transmission of Aeromonas liquefaciens. J Fish Res Board Can. 1969;26(9):2311–8.

[58] Aquabaz Marketing. PolyAqua® product specification and information [Internet]. Caesarea: Aquabaz Marketing; c2020 [cited 2020 Jul 17]. Available from: https://aquabaz.tripod.com/polyaqua.htm.

[59] Kordon LLC. PolyAqua® Professional Fish Protector™ product description [Internet]. Hayward: Kordon LLC; c2010 [cited 2023 Apr 18]. Available from: http://www.kordon.com/kordon/products/water-conditioners/novaqua#compatabilities-contraindications!

[60] Ficke A. D. & Myrick C. A. A method for monitoring movements of small fishes in urban streams. N Am J Fish Manag. 2009;29(5):1444–53.

[61] Hockersmith E, Brown RS and Liedtke TL. Comparative performance of acoustic-tagged and passive integrated transponder-tagged juvenile salmonids. Seattle (WA): Northwest Fisheries Science Center, Fish Ecology Division; February 2008 Report. Contract No.: W66QKZ60441152. Sponsored by U.S. Army Corps of Engineers, Portland District.

[62] Seitz AC, Norcross BL, Payne JC, Kagley AN, Meloy B, Gregg JL et al. Feasibility of surgically implanting acoustic tags into Pacific herring. Trans Am Fish Soc. 2010;139(5):1288–91.

[63] Gaines PC, Martin CD. Feasibility of dual-marking age-0 Chinook salmon for mark-recapture studies. N Am J Fish Manag. 2004;24:1456–9.

[64] Mueller RP, Moursund RA, Bleich MD. Tagging pacific juvenile lamprey with passive integrated transponders: methodology, short-term mortality, and influence on swimming performance. N Am J Fish Manag. 2006;26(2):361–6.

[65] Hockersmith EE, Muir SG, Smith BP, Sandford BP, Adams NS Plumb JM et al. Comparative performance of sham radio-tagged and pit-tagged juvenile salmon. Seattle (WA): Northwest Fisheries Science Center, Fish Ecology Division; December 2000 Report. Contract No.: W66QKZ91521282. Sponsored by U.S. Army Corps of Engineers, Portland District.

[66] Office of Laboratory Animal Welfare. U.S. government principles for the utilization and care of vertebrate animals used in testing, research, and training [Internet]. Bethesda (MD): National Institute of Health; c2015 [cited 2023 Jan 25]. Available from: https://olaw.nih.gov/policies-laws/phs-policy.htm#USGovPrinciples.

[67] Marsh DM, Harmon JR, McIntyre KW, Thomas KL, Paasch NN, Sandford BP et al. Research related to transportation of juvenile salmonids on the Columbia and Snake Rivers. Seattle (WA): Northwest Fisheries Science Center, Fish Ecology Division; 1996 Report. Delivery Order No.: E86960099. Sponsored by U.S. Army Corps of Engineers, Walla Walla District.

[68] Marsh DM, Harmon JR, Paasch NN, Thomas KL, McIntyre KW, Sandford BP et al. Research related to transportation of juvenile salmonids on the Columbia and Snake Rivers, 2000. Seattle (WA): Northwest Fisheries Science Center, Fish Ecology Division; 2001 Report. Delivery Order No.: E86960099. Sponsored by U.S. Army Corps of Engineers, Walla Walla District.

[69] Summerfelt RB, Smith LS. Anesthesia, surgery, and related techniques. In: Schreck CB, Moyle PB, editors. Methods for fish biology. Bethesda (MD): American Fisheries Society; 1990. p. 213–72.

[70] Winston JR. Fish health management. In: 2nd ed. Wedemeyer GA, editor. Fish hatchery management. Bethesda (MD): American Fisheries Society; 2001. p. 559–40.

[71] Austin B, Austin DA. Bacterial fish pathogens: disease of farmed and wild fish. 3rd ed. New York: Springer; 1999.

[72] Tukey J. Comparing individual means in the analysis of variance. Biometrics. 1949;5(2):99–14. 18151955

[73] Hosmer DW, Lemeshow S, May S. Applied survival analysis: regression modeling of time to event data. 2nd ed. Hoboken: Wiley & Sons; 2008.

[74] Lawless JF. Statistical models and methods for lifetime data. New York: Wiley & Sons; 1982.

[75] Zuur A, Leno EN, Walker N, Saveliev AA, Smith GM. Mixed effects models and extensions in ecology with R. New York: Springer-Verlag; 2009.

[76] Burnham KP, Anderson DR. Model selection and multimodel inference: a practical information-theoretic approach. 2nd ed. New York: Springer-Verlag; 2002.

[77] R Core Team. R: A language and environment for statistical computing [software]. Vienna: R Foundation for Statistical Computing; 2021 [cited 2023 May 31]. URL https://www.R-project.org/. Package MuMIn. Available from: https://cran.r-project.org/package=MuMIn.

[78] Cooke SJ, Schreer JF, Wahl DH, Philipp DP. Physiological impacts of catch-and-release angling practices on largemouth bass and smallmouth bass. In: Phillip DP, Ridgeway MS, editors. Black bass: ecology, conservation, and management: American Fisheries Society, Symposium 31; Bethesda (MD): American Fisheries Society; 2002. p. 489–12.

[79] Aksoy A, Caglayan F, Cakmak M, Apan TZ, Gocmen JS, Cakmak A. et al. An investigation of the factors that affect surgical hand disinfection with polyvidone iodine. J Hosp Infect. 2005 Sep;61(1):15–9. doi: 10.1016/j.jhin.2005.01.015 . 16002180

[80] Booth HW. Suture materials and tissue adhesives. In: Slatter DH II, von Recum AF, editors. Textbook of small animal surgery. Philadelphia (PA): W. B. Saunders Company; 1985. p. 334–44.

[81] Gilliland ER. Comparison of absorbable sutures used in largemouth bass liver biopsy surgery. Prog Fish Cult. 1994;56(1):60–1.

[82] Thoreau X, Baras E. Evaluation of surgery procedures for implanting telemetry transmitters into the body cavity of tilapia Oreochromis aureaus. Aquat Living Resour. 1997;10:207–11.

[83] Wagner GN, Stevens ED, Byrne P. Effects of suture type and patterns on surgical wound healing in rainbow trout. Trans Am Fish Soc. 2000;129(5):1196–205.

[84] Hurty CA, Brazic DC, Law JM, Sakamoto K, Lewbart GA. Evaluation of the tissue reactions in the skin and body wall of koi (Cyprinus carpio) to five suture materials. Vet Rec. 2002;151(11):324–8. doi: 10.1136/vr.151.11.324 12356236

[85] Govett PD, Harms CA, Linder KE, Marsh JC, Wyneken J. Effect of four different suture materials on the surgical wound healing of loggerhead sea turtles, Caretta caretta. J Herpetol Med Surg. 2004;14(4):6–10.

[86] Tuttle AD, Law JM, Harms CA, Lewbart GA, Harvey SB. Evaluation of the gross and histologic reactions to five commonly used suture materials in the skin of the African clawed frog (Xenopus laevis). J Am Assoc Lab Anim Sci. 2006;45(6):22–6. 17089987

[87] Jepsen N, Mikkelsen JS, Koed A. Effects of tag and suture type on survival and growth of brown trout with surgically implanted telemetry tags in the wild. J Fish Biol. 2008;72:594–02.

[88] Nemetz TG, MacMillan JR. Wound healing of incisions closed with a cyanoacrylate adhesive. Trans Am Fish Soc. 1988;117(2):190–5.

[89] Petering RW, Johnson DL. Suitability of a cyanoacrylate adhesive to close incisions in black crappies used in telemetry studies. Trans Am Fish Soc. 1991;120(4):535–7.

[90] Booth HW. Suture materials, tissue adhesives, staplers, and ligating clips. In: 3rd ed. Slatter DH, editor. Textbook of small animal surgery. Philadelphia (PA): Saunders; 2003;1. p. 235–44.

[91] Rouch JK. Biomaterials and surgical Implants. In: 3rd ed. Slatter DH, editor. Textbook of small animal surgery. Philadelphia (PA): Saunders; 2003;1. p. 141–8.

[92] Knights BC, Lasee BA. Effects of transmitters on adult bluegills at two temperatures. Trans Am Fish Soc. 1996;125(3):440–9.

[93] Walsh MG, Bjorgo KA, Isely J. Effects of implantation method and temperature on mortality and loss of simulated transmitters in hybrid striped bass. Trans Am Fish Soc. 2000;129(2):539–44.

[94] Columbia Basin Fish and Wildlife Authority. PIT tag marking procedures manual, version 2.0.; Report. Portland (OR): Columbia Basin Pit Tag Steering Committee; 1999.

[95] Nelle P, Ward MB. A field manual of scientific protocols for capture, handling, and tagging of wild salmonids in the upper Columbia River Basin using Passive Integrated Transponder (PIT) tags within the upper Columbia monitoring strategy. Wauconda (WA): Terraqua, Incorporated; 2008 Report. Sponsored by Bonneville Power Administration, Integrated Status and Effectiveness Monitoring Program.

[96] Monan GE, Johnson JH, Esterberg G. Electronic tags and related tracking techniques aid in study of migrating salmon and steelhead trout in the Columbia River basin. Mar Fish Rev. 1975;37(2):9–15.

[97] Jenkins JA, Bart HL Jr, Bowker JD, Bowser PR, MacMillan JR, Nickum JG, et al. Guidelines for use of fishes in research: revised and expanded. Bethesda (MD): American Fisheries Society; 2014.

