# Supplementary figures and images for: Evaluation of healing progression at surgical incision sites and the use of antiseptics for enhancing post-operative survival in subyearling Chinook salmon (Oncorhynchus tshawytscha)
Source: PLoS One. 2023 Jul 20;18(7):e0288056. doi: 10.1371/journal.pone.0288056 (PMC10358896; doi:10.1371/journal.pone.0288056)

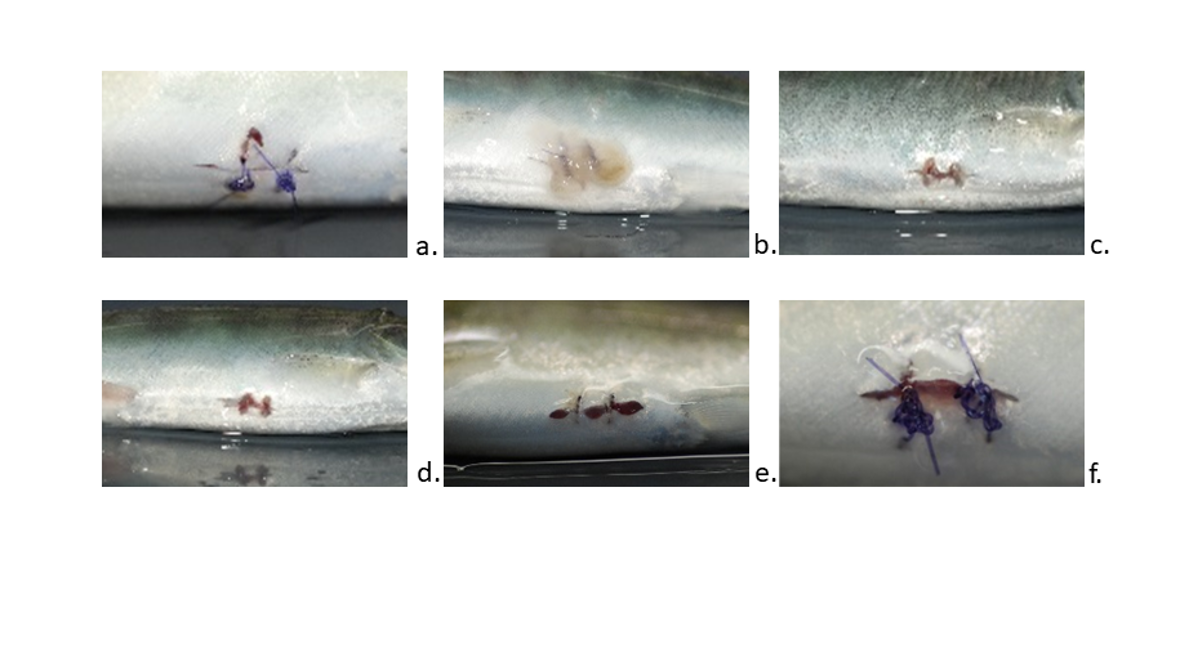

Supplement: S1 Fig — Examples of select metrics used to evaluate progression of healing including a.) suture tearing b.) presence of foreign material c.) inflammation associated with suture entrance/exit sites d.) ulceration at suture entrance/exit sites e.) incision apposition <50% of length, and f.) inflammation associated with >50% of incision length. (TIF) [file pone.0288056.s001.tif]

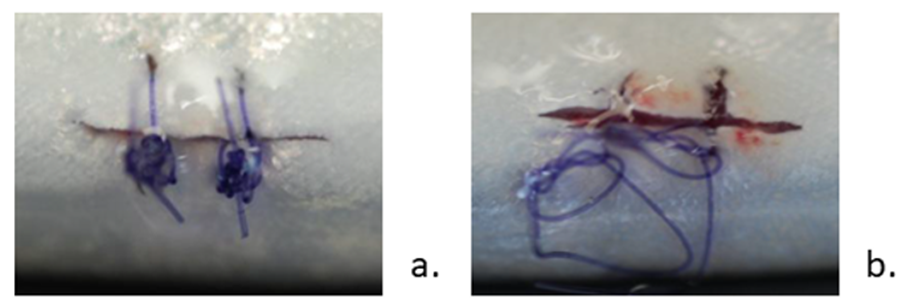

Supplement: S2 Fig — Photos from a surgeon with low survival relative to cohorts (a) and a surgeon with high survival relative to cohorts (b). Both photos were taken at the 7-d exam. Note the knots tied by the surgeon with low survival have remained intact over the first 7 d, as would be expected with proper knot tying technique. In comparison, knots tied by the surgeon with higher relative survival have loosened, with sutures pulling free from the incision. Photo b also illustrates suture tearing and the resulting wound perpendicular to the incision. (TIF) [file pone.0288056.s002.tif]
