# Supplementary material for: Evaluation of healing progression at surgical incision sites and the use of antiseptics for enhancing post-operative survival in subyearling Chinook salmon (Oncorhynchus tshawytscha)
Source: PLoS One. 2023 Jul 20;18(7):e0288056. doi: 10.1371/journal.pone.0288056 (PMC10358896; doi:10.1371/journal.pone.0288056)
Supplement: S1 Table — The percentage of fish that died before 28 d is in parentheses. (DOCX) [file pone.0288056.s003.docx]

Table S1. Number of fish tagged by surgeon and replicate. The percentage of fish that died before 28 d is in parentheses.

|  |  |  |  |  |  |  |  |  |  |  |
| --- | --- | --- | --- | --- | --- | --- | --- | --- | --- | --- |
|  | **Number of fish tagged by each surgeon (% mortality)** | | | | | | | | | |
| **Surgeons** | **1** | **2** | **3** | **4** | **5** | **6** | **7** | **8** | **Total** | **Fish that died prior to 28 d** |
| **A** |  |  |  |  |  |  |  |  |  |  |
| **B** |  | 63 (14.3) |  | 64 (39.7) |  |  | 64 (53.4) | 64 (68.9) | 255 | 107 |
| **C** | 64 (10.9) | 65 (16.9) | 65 (32.3) | 65 (33.3) | 64 (33.3) | 64 (21.9) |  | 64 (53.1) | 451 | 129 |
| **D** | 60 (10.0) | 62 (17.7) | 64 (31.3) |  | 64 (25.8) | 64 (21.9) | 64 (32.2) | 64 (57.7) | 442 | 120 |
| **E** | 63 (20.6) | 62 (18.3) |  |  | 64 (41.3) | 63 (25.4) |  | 64 (57.1) | 316 | 102 |
| **F** | 65 (26.2) | 64 (17.5) | 64 (49.2) | 63 (48.4) | 64 51.6) |  |  |  | 320 | 122 |
| **G** |  |  |  | 65 (51.6) |  |  | 64 (70.6) |  | 129 | 69 |
| **H** |  |  | 65 (47.9) |  |  | 64 (28.1) | 64 (55.2) | 64 (79.4) | 257 | 131 |
| **I** |  |  |  | 63 (51.6) | 64 (39.1) |  |  |  | 127 | 57 |
| **Other*** | 67 (19.4) | 4 (0.0) | 62 (42.9) |  |  | 66 (27.7) | 63 (40.3) |  | 262 | 83 |

* Combined rank for four surgeons who tagged on a periodic basis.
