# Supplementary material for: Evaluation of healing progression at surgical incision sites and the use of antiseptics for enhancing post-operative survival in subyearling Chinook salmon (Oncorhynchus tshawytscha)
Source: PLoS One. 2023 Jul 20;18(7):e0288056. doi: 10.1371/journal.pone.0288056 (PMC10358896; doi:10.1371/journal.pone.0288056)
Supplement: S2 Table — Mean and overall rank scores are also listed; a lower ranking corresponds with higher mortality. (DOCX) [file pone.0288056.s004.docx]

Table S2. Rank among surgeons based on percentage mortality for study replicates 1-8. Mean and overall rank scores are also listed; a lower ranking corresponds with higher mortality.

|  |  |  |  |  |  |  |  |  |  |  |
| --- | --- | --- | --- | --- | --- | --- | --- | --- | --- | --- |
|  | **Mortality rank (1-5) among surgeons by replicate** | | | | | | | | | |
| **Surgeons** | **1** | **2** | **3** | **4** | **5** | **6** | **7** | **8** | **Mean** | **Overall ranking** |
| **A** |  |  |  |  |  |  |  |  |  |  |
| **B** |  | 2 |  | 2 |  |  | 2 | 2 | 2.0 | 3 |
| **C** | 3 | 1 | 3 | 3 | 3 | 3 |  | 4 | 2.8 | 4 |
| **D** | 3 | 1 | 3 |  | 4 | 3 | 4 | 3 | 3.0 | 4 |
| **E** | 2 | 1 |  |  | 2 | 2 |  | 3 | 2.0 | 2 |
| **F** | 1 | 1 | 1 | 1 | 1 |  |  |  | 1.0 | 1 |
| **G** |  |  |  | 1 |  |  | 1 |  | 1.0 | 1 |
| **H** |  |  | 1 |  |  | 1 | 2 | 1 | 1.3 | 1 |
| **I** |  |  |  | 1 | 2 |  |  |  | 1.5 | 2 |
| **Other*** | 2 | 3 | 2 |  |  | 1 | 3 |  | 2.2 | 2 |

* Combined rank for four surgeons who tagged on a periodic basis.
