# Supplementary material for: Evaluation of healing progression at surgical incision sites and the use of antiseptics for enhancing post-operative survival in subyearling Chinook salmon (Oncorhynchus tshawytscha)
Source: PLoS One. 2023 Jul 20;18(7):e0288056. doi: 10.1371/journal.pone.0288056 (PMC10358896; doi:10.1371/journal.pone.0288056)
Supplement: S3 Table — (DOCX) [file pone.0288056.s005.docx]

Table S3. Number of fish that died prior to being ponded at the Bonneville Dam juvenile fish monitoring facility, by treatment and tagging date (%).

|  |  |  | **Peroxide (mg/L)** | | | **Salt (ppt)** | |  |
| --- | --- | --- | --- | --- | --- | --- | --- | --- |
| **Date** | **Reference** | **Argent** | **25** | **50** | **100** | **10** | **30** | **PolyAqua** |
| 3-Jun | 0 (0) | 0 (0) | 0 (0) | 0 (0) | 0 (0) | 0 (0) | 0 (0) | 0 (0) |
| 5-Jun | 0 (0) | 0 (0) | 0 (0) | 5 (2) | 0 (0) | 0 (0) | 0 (0) | 0 (0) |
| 10-Jun | 0 (0) | 0 (0) | 0 (0) | 0 (0) | 2.5 (1) | 0 (0) | 0 (0) | 0 (0) |
| 12-Jun | 0 (0) | 0 (0) | 5 (2) | 7.5 (3) | 0 (0) | 0 (0) | 2.5 (1) | 0 (0) |
| 18-Jun | 0 (0) | 0 (0) | 0 (0) | 2.5 (1) | 5 (2) | 0 (0) | 0 (0) | 0 (0) |
| 20-Jun | 0 (0) | 0 (0) | 0 (0) | 0 (0) | 0 (0) | 0 (0) | 0 (0) | 0 (0) |
| 27-Jun | 2.5 (1) | 0 (0) | 2.5 (1) | 30 (12) | 32.5 (13) | 0 (0) | 0 (0) | 10 (4) |
| 1-Jul | 0 (0) | 2.5 (1) | 5 (2) | 2.5 (1) | 5 (2) | 0 (0) | 2.5 (1) | 0 (0) |
| 10-Jul | 0 (0) | 0 (0) | 5 (2) | 2.5 (1) | 12.5 (5) | 0 (0) | 0 (0) | 2.5 (1) |
| 16-Jul | 0 (0) | 7.5 (3) | 10 (4) | 10 (4) | 22.5 (9) | 0 (0) | 2.5 (1) | 0 (0) |
| Total | 0.2 (1) | 1 (4) | 2.8 (11) | 6 (24) | 8 (32) | 0 (0) | .8 (3) | 1.3 (5) |
| Reps 1-8 | .3 (1) | .3 (1) | 1.6 (5) | 6 (19) | 5.6 (18) | 0.00 | 0.6 (2) | 1.3 (4) |
